# The effect of preoperative pelvic floor muscle training on urinary and colorectal-anal distress in women undergoing pelvic organ prolapse surgery—a randomized controlled trial

**DOI:** 10.1007/s00192-021-04684-3

**Published:** 2021-02-13

**Authors:** Seema Mathew, Maria Øyasæter Nyhus, Øyvind Salvesen, Kjell Åsmund Salvesen, Signe Nilssen Stafne, Ingrid Volløyhaug

**Affiliations:** 1 Department of Obstetrics and Gynecology, St. Olavs University Hospital, Trondheim, Norway; 2 Department of Clinical and Molecular Medicine, Norwegian University of Science and Technology, Trondheim, Norway; 3 Department of Public Health and Nursing, Norwegian University of Science and Technology, Trondheim, Norway; 4 Clinical Services, St. Olavs University Hospital, Trondheim, Norway

**Keywords:** Randomized clinical trial, Pelvic floor, Muscle training, Pelvic organ prolapse, Urinary incontinence, Fecal incontinence

## Abstract

**Introduction and hypothesis:**

Pelvic floor muscle training (PFMT) improves urinary incontinence and mild pelvic organ prolapse (POP). We aimed to investigate the effect of preoperative PFMT on urinary and colorectal-anal distress and related quality of life (QoL) in women with severe POP scheduled for surgery.

**Methods:**

Randomized controlled trial of 159 women scheduled for POP surgery (intervention = 81, controls = 78). Intervention consisted of daily PFMT from inclusion to the day of surgery. Symptoms and QoL were assessed at inclusion, day of surgery and 6 months postoperatively using the Urinary Distress Inventory (UDI-6), Colorectal-Anal Distress Inventory (CRADI-8), Urinary Impact Questionnaire (UIQ) and Colorectal-Anal Impact Questionnaire (CRAIQ) (range 0–100). Mixed model statistical analyses were used.

**Results:**

One hundred fifty-one (95%) women completed the study (intervention = 75, controls = 76). Mean waiting times until surgery and follow-up were 22 and 28 weeks. There was no difference in mean postoperative symptom and QoL scores (95% CI) between the intervention and control group: UDI-6 16 (12–21) vs. 17 (13–22), CRADI-8 15 (11–18) vs. 13 (10–16), UIQ 11 (7–15) vs. 10 (6–13) and CRAIQ 5 (2–7) vs. 6 (4–9), all *p* > 0.05. Overall mean scores were reduced from baseline to postoperative follow-up: UDI-6 37 (33–41) vs. 17 (14–20), CRADI-8 22 (19–25) vs. 14 (11–16); UIQ 28 (24–32) vs. 10 (7–13) and CRAIQ 16 (12–19) vs. 5 (3–7), all *p* < 0.01.

**Conclusions:**

We found no added effect of preoperative PFMT on symptoms or QoL related to urinary and colorectal-anal distress in women scheduled for POP surgery. They achieved symptomatic improvement postoperatively regardless of PFMT.

**Clinical trial registration:**

The study was registered in clinicaltrials.gov: NCT 03,064,750.

## Introduction

Urinary and colorectal-anal distress has a negative impact on quality of life [1–3]. These symptoms are highly prevalent in women with pelvic organ prolapse (POP) because of shared risk factors such as age, parity and pelvic floor trauma occurring during delivery [1–5]. Injury to nerves, connective tissue and muscles contributes to the pathophysiology of pelvic floor disorders [1, 6]. Strengthening the pelvic floor muscles is therefore one option to treat pelvic floor disorders [7].

Intensive pelvic floor muscle training (PFMT) is effective in treating stress urinary incontinence and symptomatic mild POP, reducing bulge sensation and frequent urination [8, 9]. PFMT is also effective in treating anal incontinence symptoms and improve quality of life, but the effect on other urinary symptoms or colorectal-anal symptoms such as emptying difficulties is unclear [9–13]. Repeated contractions improve the strength and endurance of the pelvic floor muscles, providing better support to pelvic organs and improving urinary continence [9, 12]. However, most studies have either examined women in the immediate postpartum period or women with isolated stress urinary incontinence [10, 12]. Other studies of women undergoing POP surgery have mainly focused on the effect of peri- or postoperative PFMT on urinary and colorectal-anal symptoms, and one study found marginal effects of PFMT on quality of life [14–17]. Previous studies with < 100 participants have included women scheduled for surgery because of different conditions (POP, urinary incontinence and hysterectomy for other reasons), and it is unclear whether the positive effect of peri- and postoperative PFMT was found in women with POP [15, 16, 18]. Any additional effect of preoperative PFMT on urinary and colorectal-anal symptoms and quality of life in women with advanced POP has not been thoroughly investigated.

Our aim was therefore to examine the effect of preoperative PFMT on urinary and colorectal-anal symptoms in women scheduled for POP surgery. We also aimed to study any effect on quality of life related to these symptoms.

## Materials and methods

This was a randomized controlled trial (RCT) of women scheduled for POP surgery at Trondheim University Hospital, Norway, from January 2017 through March 2019. Women were recruited from the outpatient urogynecological clinic from January 2017 through June 2018. All participants signed a written informed consent form at a preoperative consultation. Inclusion criteria were indication for POP surgery (bulge sensation and POP stage ≥ 2), age > 18 years and fluent in Norwegian or English. Women declining participation, needing immediate surgery or with cognitive impairments were excluded. The study was approved by the Regional Committee for Medical and Health Research Ethics (REK2015/1751/midt) and registered in clinicaltrials.gov with the identifier NCT 03,064,750.

Age, parity, delivery mode, height, weight, smoking habits, menopausal status, hormonal therapy, pessary use and any previous PFMT or POP surgery were registered at inclusion. Surgical procedure was determined according to the clinical practice considering age, prolapse grade, involved compartments and any previous POP surgery. Available procedures were: colporrhaphy (anterior and posterior), perineoplasty, enterocele correction, cervical amputation with shortening of the ligaments, vaginal hysterectomy, sacrospinous ligament fixation, laparoscopic robot-assisted sacrouteropexy or sacrocolpopexy. The procedures performed and any surgical complications were registered.

At inclusion, women were randomized to intervention or control with the allocation ratio of 1:1 and stratified using POP stage > or < 3 and age > or < 60 years using a web-based randomization tool (WebRAND). Participants were examined and patient-reported outcomes collected at inclusion, day of surgery (minimum 3 months later) and 6 months postoperatively by one of three authors (SM/MØN/IV). Data were registered in a web-based case report form (WebCRF) provided by the Unit of Applied Clinical Research, Norwegian University of Science and Technology. A gynecological examination was performed with the participant in the supine position with hips and knees semi-flexed and abducted. POP was assessed at maximum Valsalva according to the Pelvic Organ Prolapse Quantification (POP-Q) system [19]. Examiners were not blinded to background data or group allocation at examination. At each visit the women answered a validated Norwegian translation of the Pelvic Floor Distress Inventory (PFDI-20) and Pelvic Floor Impact Questionnaire (PFIQ-7) [20, 21]. For quantification of urinary and colorectal-anal distress and impact on quality of life, we used the PFDI-20 sub-scales: Urinary Distress Inventory (UDI-6) and Colorectal Anal Distress Inventory (CRADI-8) and PFIQ-7 subscales: Urinary Impact Questionnaire (UIQ) and Colorectal Anal Impact Questionnaire (CRAIQ), all with a range of 0–100 where 100 is the worst bother [20, 21]. The average waiting time to surgery at Trondheim University Hospital during the study period was 3 months. Waiting time was not influenced by group allocation.

Women allocated to intervention received written information regarding the correct pelvic floor exercise technique at inclusion. They were given written lifestyle advice regarding diet and proper emptying of the bladder and bowel as well as instructions on contraction of the pelvic floor muscles when sneezing, coughing or laughing [7, 9]. Vaginal examination was performed by one of the examiners (SM, MØN, IV) at inclusion and by a pelvic floor physiotherapist at visits 2 and 6 weeks after inclusion to ensure proper contraction for women in the intervention group. Women were instructed to perform intensive pelvic floor muscle exercise with 8–12 maximal contractions holding at least 6–8 s three times daily from time of inclusion until the day of surgery [22, 23]. They were informed about voluntary weekly group training sessions at the baseline examination and at the first consultation with the physiotherapist 2 weeks after inclusion. They were required to record daily exercises in a training diary, to be handed in at the day of surgery. Women who failed to deliver a training diary were interviewed by telephone regarding the number of days per week they had performed training and the number of repetitions each day. A ≥ 70% completion of daily exercise rate was defined as adherence to the protocol [24, 25]. Women in the control group received no intervention in the waiting time for surgery. All postmenopausal women, regardless of randomization, received local estrogen therapy unless contraindicated.

Primary outcome measures of the RCT were pelvic floor muscle strength assessed by palpation and ultrasound and symptoms of pelvic floor disorders as registered in clinicaltrials.gov (NCT 03,064,750). We have previously reported results regarding muscle contraction assessed by palpation, manometry and ultrasound as well as prolapse symptoms [25]. In the present article, we report on another of the primary outcomes: symptoms of urinary and colorectal-anal distress assessed by validated PFDI sub-scales: UDI-6 and CRADI-8. A secondary outcome was patient reported quality of life related to urinary and colorectal-anal symptoms using the PFIQ sub-scales: UIQ and CRAIQ.

Sample size calculation was based on differences in pelvic floor muscle contraction. A mean modified Oxford scale of 2.6 ± 1.3 was anticipated and a clinically relevant change in modified Oxford scale at 6-month follow-up of 3.2 ± 1.3. With power 80%, *p* = 0.05 and sampling ratio 1:1, a study sample of 74 women in each group was considered sufficient.

### Statistical methods

Outcomes were analyzed following an intention-to-treat principle. We used IBM SPSS Statistics version 25 (SPSS Inc., Chicago, IL) and R version 3.6.3 (R Project for Statistical Computing) to perform statistical analyses. The level of statistical significance was set at 5%. Normality of the continuous variables (UDI-6, CRADI-8, UIQ and CRAIQ) was assessed using histograms and QQ plots. Independent sample t-test was used to examine any differences between women accepting and declining randomization. Symptoms and quality of life in the intervention group versus the control group at the day of surgery and postoperative control were evaluated with mixed models analysis with a five-level combined variable for time and group status as fixed effects (baseline for total study population, day of surgery for intervention group, day of surgery for control group, postoperative follow-up intervention group and postoperative follow-up control group). The model was fitted by restricted maximum likelihood estimation and unstructured covariance for the repeated measurements of each participant. The effect of the stratification variables (POP stage > or < 3 and age > or < 60) was tested, and no effect was found. The change in the total study population with time as fixed effect (baseline for total study population, postoperative follow-up for total population) was also tested using a mixed models analysis fitted by restricted maximum likelihood estimation and unstructured covariance for the repeated measurements.

## Results

**Fig. 1:**
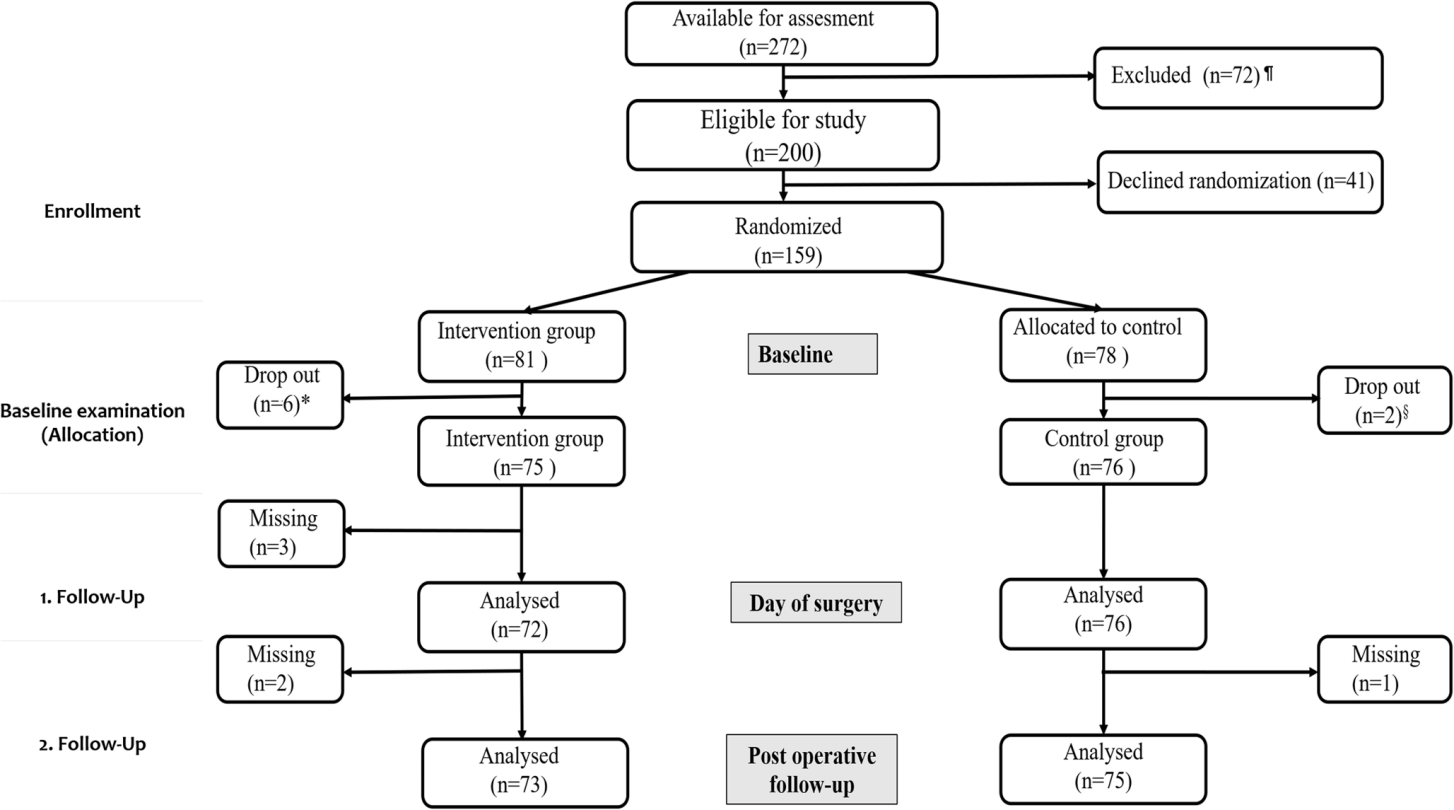
Flowchart of study population. ^¶^Declined participation (*n* = 36), missed for recruitment (*n* = 4), did not meet eligibility criteria (*n* = 32). ^*^Three women postponed surgery (one because of other medical conditions and two because of symptomatic improvement). Three women declined further participation. ^§^Two women postponed surgery because of improvement of symptoms

During the recruitment period from January 2017 through June 2018, 272 women were referred for POP surgery. One hundred thirteen women were excluded because they refused participation, did not fulfill the inclusion criteria or declined randomization; see the flow chart (Fig. 1). Of the 159 randomized women, 151 (95%) completed the study, 75 in the intervention group and 76 in the control group. Data collection ended in June 2019.

Background characteristics and outcome variables are outlined in Table 1. Overall, 92/151 (61%) had POP stage ≥ 3. The proportion of women undergoing an isolated anterior or posterior compartment repair was 28/151 (19%) and 27/151 (18%), respectively. Thirty-eight (25%) women had an isolated central compartment repair. A combination of procedures involving more than one compartment was performed in 58/151 (38%) women. Sixty (80%) women in the training group achieved an adherence level of ≥ 70% to the intervention. None of the participants met for the voluntary weekly group training sessions. Women declining randomization were similar to study participants in POP stage ≥ 3, body mass index and parity, but significantly older compared to the study participants (67 vs. 61 years, *p* = 0.002).

**Table 1:** Participant demographics and main findings for the intervention and control groups

	Intervention group *N* = 75	Control group *N* = 76
Demographics		
	Mean (SD)	
Age (years)	60.1 (11.2)	60.6 (10.9)
Body mass index (kg/m^2^)	26.3 (4.4)	25.7 (4.1)
Parity (number)	2.3 (0.8)	2.6 (0.9)
Waiting time before surgery (weeks)	21.6 (8.5)	23.2 (10.8)
Time to postoperative follow-up (weeks)	28.7 (8.0)	27.6 (7.6)
	*N* (%)	
Normal vaginal delivery	51 (68.0)	55 (72.4)
Operative vaginal delivery (including breech or twin delivery)	22 (29.3)	20 (26.3)
Smoking	10 (13.9)^*^	6 (7.9)
Postmenopausal	59 (79.7)^*^	59 (77.6)
Local estrogen therapy	47 (63.5)^*^	48 (63.2)
Previous pessary use	50 (67.6)^*^	60 (78.9)
Previous pelvic floor muscle training	13 (17.6)^*^	14 (18.4)
Previous pelvic organ prolapse surgery	7 (9.5)^*^	11 (14.5)
Objective findings		
Pelvic organ prolapse quantification (POPQ) ≥ 3	44 (58.7)	48 (63.2)
Subscale scores at inclusion (range 0–100)	Mean (95% CI)	
Urinary distress inventory (UDI-6)	38.0 (32.7–43.2)^*^	35.5 (29.2–41.8)^§^
Colorectal-anal distress inventory (CRADI-8)	23.9 (20.3–27.4)	20.4 (16.1–24.6)^¶^
Urinary impact questionnaire (UIQ)	30.2 (24.0–36.3)	25.5 (19.5–31.6)^**^
Colorectal anal impact questionnaire (CRAIQ)	18.8 (13.4–24.2)	12.8 (8.0–17.6)^**^

^*^Data missing for one participant, ^**^data missing for two participants, ^¶^data missing for four participants, ^§^data missing for six participants

**Fig. 2:**
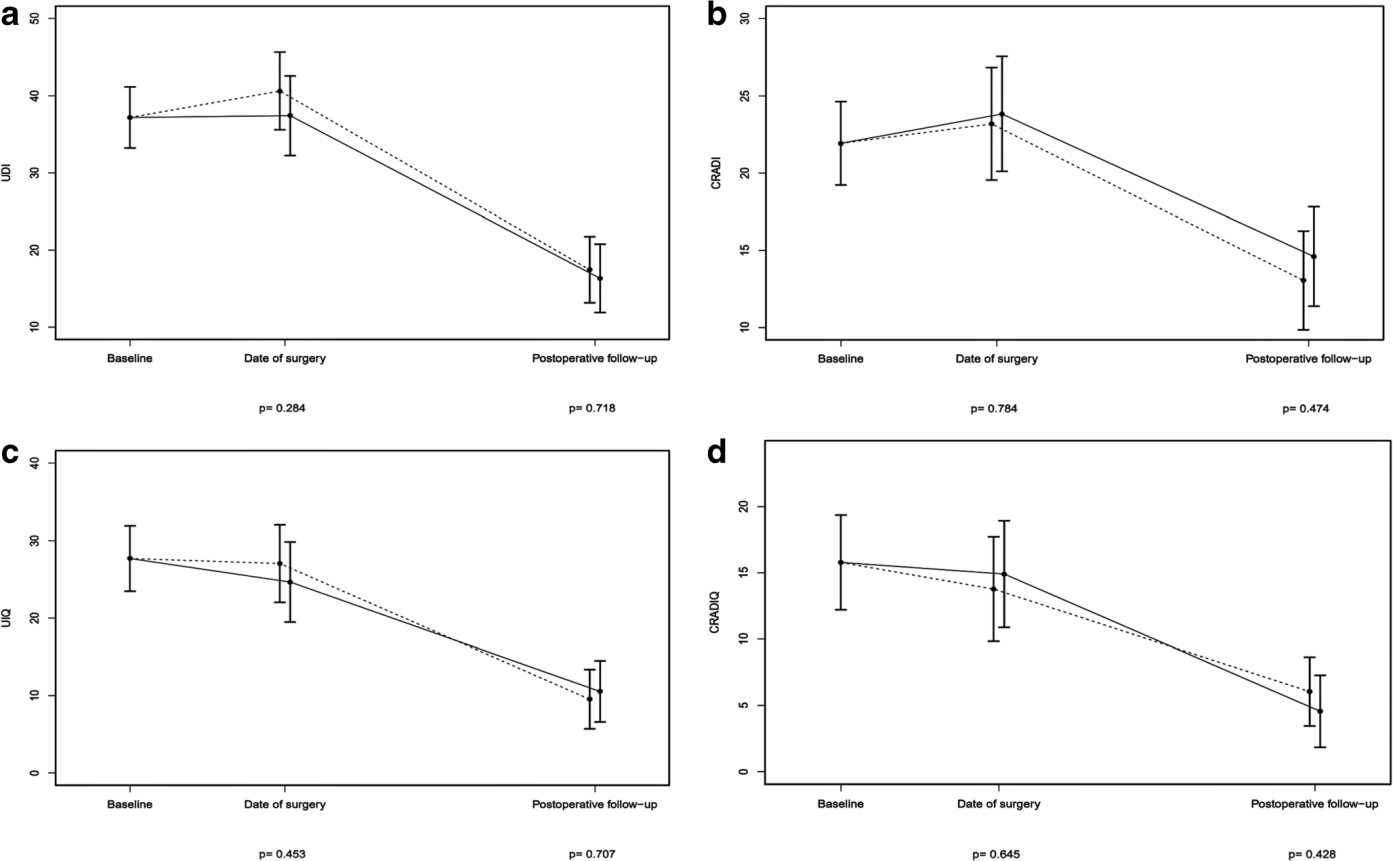
Figure comparing symptoms and related quality of life in the intervention group (solid line) and the control group (dashed line) from baseline to the day of surgery and the postoperative follow-up, using linear mixed models analysis. Examination time (baseline, day of surgery and postoperative follow-up) on the x-axis and mean score with 95% confidence interval on the y-axis of the **a** urinary distress inventory (UDI-6), **b** colorectal-anal distress inventory (CRADI-8), **c** urinary impact questionnaire (UIQ) and **d** colorectal-anal impact questionnaire (CRAIQ)

Mean (SD) and median (range) waiting time to surgery was 22 (10) and 21(7–84) weeks, and women were examined postoperatively after mean 28 (8) and median 26 (11–79) weeks. There was no statistically significant difference in UDI-6 or CRADI-8 scores or change in scores between intervention and control groups at day of surgery or postoperatively; see Table 2. Analysis of the quality of life related to urinary and colorectal-anal distress (UIQ and CRAIQ) revealed similar findings (Table 2). Figure 2 demonstrates the linear mixed model analysis of the change in scores for the intervention and control group at each examination. Overall, there was a statistically significant decrease in symptoms and improvement in quality of life from baseline to postoperative control in the total study population (Table 3).

**Table 2:** Mean values with 95% confidence intervals (CI) at baseline, day of surgery and postoperative follow-up. Mean differences with 95% CI between intervention and control groups

	Baseline^*^	Day of surgery^**^				Postoperative follow-up^***^			
	*n* = 151	Intervention *n* = 72	Control *n* = 76	Difference between groups^¶^		Intervention *n* = 73	Control *n* = 75	Difference between groups^¶^	
	Mean (95% CI)	Mean (95% CI)		Mean (95% CI)	*p*	Mean (95% CI)	Mean (95% CI)	Mean (95% CI)	*p*
*Symptoms subscales (range 0–100)*									
Urinary distress inventory (UDI-6)	37.2 (33.2–41.2)	37.4 (32.3–42.6)	40.6 (35.6–45.6)	-3.2 (-9.1–2.7)	0.284	16.3 (11.9–20.8)	17.4 (13.2–21.7)	-1.1 (-7.2–4.9)	0.718
Colorectal anal distress inventory (CRADI-8)	21.9 (19.2–24.6)	23.8 (20.1–27.5)	23.2 (19.6–26.8)	0.6 (-4.0–5.3)	0.784	14.6 (11.4–17.8)	13.1 (9.9–16.2)	1.5 (-2.7–5.8)	0.474
*Quality of life subscales (range 0–100)*									
Urinary impact questionnaire (UIQ)	27.7 (23.5–31.9)	24.7 (19.5–29.8)	27.0 (22.0–32.1)	-2.4 (-8.7–3.9)	0.453	10.5 (6.6–14.5)	9.5 (5.7–13.4)	1.0 (-4.2–6.2)	0.707
Colorectal anal impact questionnaire (CRAIQ)	15.8 (12.2–19.4)	14.9 (10.9–18.9)	13.8 (9.8–17.7)	1.1 (-3.7–6.0)	0.645	4.6 (1.8–7.3)	6.0 (3.5–8.6)	-1.4 (-5.2–2.2)	0.428

^*^Missing values UDI:7, CRADI:4, UIQ:2, CRAIQ:2

^**^Missing values, UDI:19, CRADI:17, UIQ:15, CRAIQ:15

^***^Missing values UDI:10, CRADI:12, UIQ:8, CRAIQ:10

^¶^A positive mean difference in symptoms scores (UDI-6 and CRADI-8) and quality of life parameters (UIQ and CRAIQ) indicates improvement in the control group whereas a negative mean difference indicated improvement in the intervention group

**Table 3:** Mean values, median and range for total population at baseline and postoperative follow-up, showing mean difference and 95% confidence interval (95% CI) with positive values indicating reduction of symptoms and related impact on quality of life scores postoperatively

	Baseline *n* = 151^*^		Postoperative follow-up *n* = 148^*^	Difference between baseline and postoperative follow-up	
	Mean (95% CI)	Mean (95% CI)		Mean difference (95% CI)	*p*
Symptoms- subscales (range 0–100)					
Urinary distress inventory (UDI-6)	37.2 (33.3–41.2)		16.9 (13.8–20.0)	20.3 (16.0–24.6)	< 0.001
Colorectal-anal distress inventory (CRADI-8)	21.9 (19.2–24.6)		13.8 (11.4–16.2)	8.1 (5.5–10.7)	< 0.001
Quality of life subscale range (0–100)					
Urinary impact questionnaire (UIQ)	27.7 (23.5–31.9)		10.0 (7.2–12.9)	17.7 (13.7–21.7)	< 0.001
Colorectal-anal impact questionnaire (CRAIQ)	15.7 (12.2–19.4)		5.3 (3.4–7.3)	10.4 (6.9–14.0)	< 0.001

^*^Missing values UDI:16, CRADI:16, UIQ:10, CRAIQ:12

Two major complications were registered: an intestinovaginal fistula after laparoscopic sacrocolpopexy and one postoperative hemorrhage, both requiring further surgery. Other complications were postoperative urinary tract infection requiring treatment in 3/151 (2%) and one woman (< 1%) with persisting residual urine after 6 months.

## Discussion

In this randomized controlled trial of women scheduled for POP surgery, we found no effect of preoperative PFMT on urinary or colorectal-anal distress and related quality of life 6 months after surgery. Women achieved symptomatic improvement postoperatively regardless of PFMT.

PFMT is shown to reduce stress urinary incontinence, anal incontinence and symptoms of mild POP, but there is less evidence regarding the effect of a strong and well-functioning pelvic floor on other urinary symptoms and colorectal-anal distress [8, 9, 11, 12, 17]. A systematic review on fecal incontinence in adults reported conflicting results in different studies comparing PFMT to other conservative treatments such as dietary advice, medical management and PFMT with biofeedback [11]. This review included both genders and varying treatment durations from 1 to 12 months, thus making it difficult to generalize [11]. Our results are consistent with previous studies on women with severe POP where peri- and postoperative PFMT did not alter symptoms of urinary and colorectal-anal distress [14, 15]. A recent study by Duarte et al. included a preoperative intervention period of 2 weeks and reported an overall improvement in symptoms and quality of life (using PFDI-20, PFIQ-7 and subscales) for all women scheduled for POP surgery, without clear advantage from PFMT in the intervention group, which agrees with our findings [14]. The study excluded women with previous POP surgery and covered a shorter postoperative follow-up of 90 days [14]. In contrast, 10% of the women in the current study had prior POP surgery, and they were followed 6 months to observe any durable effects. Tools used for symptom assessment differ between studies, and the intervention in the current study was exclusively preoperative whereas the intervention in most prior studies was mainly postoperative PFMT [15, 16]. McClurg et al. demonstrated a postoperative reduction of prolapse symptoms in the intervention group, but no effect on incontinence symptoms, although the participants had milder prolapses and other adjuncts such as electrical stimulation and biofeedback were also applied in addition to PFMT [16]. Incontinence and POP symptoms did not improve after PFMT alone in a study by Frawley et al., although they reported less de novo stress incontinence after PFMT [15]. However, the intervention consisted of only one supervised preoperative PFMT session followed by seven sessions over 1 year, and women scheduled for hysterectomy for other indications than POP were included [15]. A study by Jarvis et al. included women scheduled for urinary incontinence or POP surgery with 12-week follow-up and found reduced stress urinary incontinence after PFMT, but it is unclear whether the positive effect of PFMT was found only in women with isolated incontinence or also in women with POP [18].

The main clinical implication of our findings is that women scheduled for POP surgery have no additional benefit of PFMT on urinary and colorectal-anal symptoms. Women with advanced POP and complex injuries to the pelvic floor may need more supervised and intensive exercise or additional treatments such as nerve stimulation to increase strength. In a previous publication from this RCT, we found no difference in muscle strength or POP symptoms between the intervention and control groups [25]. The failure to improve pelvic floor muscle strength is a possible explanation for the lack of effect also on urinary and colorectal-anal distress. They experienced improved pelvic floor muscle contraction after surgery, which may explain the results from the present study of reduced urinary and colorectal-anal distress and improved quality of life at the postoperative follow-up [25]. With 60% of women having POP stage ≥ 3, it seems likely that advanced POP poses limitations to correct muscle contraction for sufficient clinical and subjective improvement. In addition, the large reduction in symptoms and improvement of quality of life after surgery may obscure any additional minor effect of PFMT after surgery. Latent stress urinary incontinence can appear after anterior compartment correction and may also be a reason for failing to detect any effect of PFMT on urinary distress in this cohort [26]. The etiology of urinary and colorectal-anal distress is complex and not solely dependent on weak pelvic floor muscle function [1, 4, 5, 27]. This cohort consists of women with extensive pelvic floor injuries, such as levator muscle injury, sphincter injury and nerve damage, all possibly contributing to the development and persistence of urinary or colorectal-anal symptoms [1]. Other chronic diseases and lifestyle habits also contribute to symptoms [1, 2].

The main strength of the present study was the randomized controlled design and the large study size. The intervention consisted of daily PFMT and 80% of women in the intervention group maintained ≥ 70% adherence, indicating that the training program was acceptable for most women scheduled for POP surgery. We included women with advanced POP in any compartment and those with prior POP surgery, representing a heterogenous cohort commonly encountered in urogynecological practice and further increasing the clinical relevance of this study. The intervention lasted on average 22 weeks, which should be sufficient to achieve muscle hypertrophy [8, 22, 23]. No adjunctive treatments such as biofeedback or nerve stimulation were given in order to uncover the exclusive effects of PFMT. Validated questionnaires designed for evaluating distress and quality of life related to urinary and colorectal-anal symptoms were used [20, 21]. We used mixed models statistics for assessment of symptoms over time and between the groups, making it possible to use all data available also for women with missing data at the day of surgery or postoperative follow-up.

A limitation was that we did not register the number of women with previous incontinence surgery. However, the randomization ensured similar distribution to the intervention and control group for both previous surgery and other potential confounders. Another limitation was that we did not record whether women in the control group performed PFMT, and this might dilute any possible difference between the groups. Participants were not blinded to the intervention, and therefore women in the intervention group might have scored higher on quality of life because of an expectation of improvement. Examining gynecologists were not blinded to group allocation at the day of surgery or at the postoperative follow-up, but since this study only presents patient-reported outcomes, this would not be relevant to the outcome. Women declining randomization were significantly older; hence, the results may not apply to the older segment of POP patients. No power calculation was performed for these outcome measures, but the women had symptom scores between 20–40 out of 100. Hence, with 75 and 76 women in each group we would expect to find a clinical and statistically significant difference in symptom scores after intervention if there were any effect of PFMT.

## Conclusion

In women with advanced POP scheduled for surgery, we found no added effect of preoperative PFMT on symptoms or quality of life related to urinary or colorectal-anal distress 6 months after surgery. Surgical prolapse correction decreased urinary and colorectal-anal symptoms and improved quality of life related to these symptoms. There is a need for long-term trials of intensive PFMT in women after corrective POP surgery in order to investigate the comprehensive effect on urinary and colorectal-anal distress and de novo incontinence.
